# Papillary thyroid cancer organoids harboring BRAF^V600E^ mutation reveal potentially beneficial effects of BRAF inhibitor-based combination therapies

**DOI:** 10.1186/s12967-022-03848-z

**Published:** 2023-01-09

**Authors:** Dong Chen, Xi Su, Lizhang Zhu, Hao Jia, Bin Han, Haibo Chen, Qingzhuang Liang, Chenchen Hu, Hao Yang, Lisa Liu, Peng Li, Wei Wei, Yongsheng Zhao

**Affiliations:** 1 Department of Thyroid and Breast Surgery, Peking University Shenzhen Hospital, Shenzhen, 518036 China; 2 Department of Nuclear Medicine, Peking University Shenzhen Hospital, Shenzhen, 518036 China; 3 Lewis Katz School of Medicine, Temple University, Philadelphia, PA 19122 USA

**Keywords:** Papillary thyroid cancer, Organoid, BRAF^V600E^, Combination therapy, Treatment prediction

## Abstract

**Backgrounds:**

Papillary thyroid cancer (PTC), which is often driven by acquired somatic mutations in BRAF genes, is the most common pathologic type of thyroid cancer. PTC has an excellent prognosis after treatment with conventional therapies such as surgical resection, thyroid hormone therapy and adjuvant radioactive iodine therapy. Unfortunately, about 20% of patients develop regional recurrence or distant metastasis, making targeted therapeutics an important treatment option. Current in vitro PTC models are limited in representing the cellular and mutational characteristics of parental tumors. A clinically relevant tool that predicts the efficacy of therapy for individuals is urgently needed.

**Methods:**

Surgically removed PTC tissue samples were dissociated, plated into Matrigel, and cultured to generate organoids. PTC organoids were subsequently subjected to histological analysis, DNA sequencing, and drug sensitivity assays, respectively.

**Results:**

We established 9 patient-derived PTC organoid models, 5 of which harbor BRAF^V600E^ mutation. These organoids have been cultured stably for more than 3 months and closely recapitulated the histological architectures as well as mutational landscapes of the respective primary tumors. Drug sensitivity assays of PTC organoid cultures demonstrated the intra- and inter-patient specific drug responses. BRAF^V600E^ inhibitors, vemurafenib and dabrafenib monotherapy was mildly effective in treating BRAF^V600E^-mutant PTC organoids. Nevertheless, BRAF inhibitors in combination with MEK inhibitors, RTK inhibitors, or chemotherapeutic agents demonstrated improved efficacy compared to BRAF inhibition alone.

**Conclusions:**

These data indicate that patient-derived PTC organoids may be a powerful research tool to investigate tumor biology and drug responsiveness, thus being useful to validate or discover targeted drug combinations.

**Supplementary Information:**

The online version contains supplementary material available at 10.1186/s12967-022-03848-z.

## Background

In the past few years, there has been an increased interest in patient-derived organoids (PDOs) derived from diseased human tissues. In contrast to the in vivo models of patient-derived mouse xenograft (PDX) that require large amounts of specimens and normally 4–8 months for development [1, 2], PDOs can be cultured from patient materials and expanded with high efficiency within 1 month. Although immortalized cell lines have proven valuable in the study of tumor pathogenesis, these in vitro models have the obvious drawbacks of bearing little resemblance to the parental tumors [3]. PDOs have been applied to model various cancers, e.g, colorectal [4], prostate [5], lung [6], liver [7], breast [8], pancreatic [9], endometrial [10], bladder [11], ovarian [12], esophagus [13], and gastrointestinal [14] cancers. PDOs have been used as in vitro disease models that recapitulate the pathological characteristics, genetic alterations, and heterogeneity of their corresponding primary tumors and can potentially serve as “avatars” for selecting clinical therapeutic regimen. Several studies have demonstrated that PDOs are able to precisely predict patient responses to drug and radiation treatments in the clinic [14–20].

Thyroid cancer has become the most common endocrine malignancy with an increasing incidence in recent years [21]. Histopathologically, thyroid cancer can be stratified into four categories: papillary thyroid cancer (PTC, 80–85%), follicular thyroid cancer (FTC, 10–15%), anaplastic thyroid cancer (ATC, 2–3%), and medullary thyroid cancer (MTC, 2–3%) [22]. PTC and FTC are collectively referred to as differentiated thyroid cancer (DTC). Among all types of thyroid cancer, PTC is the most common histologic type, and is characterized by the most frequent mutation of BRAF^V600E^ [22, 23]. This mutation leads to phosphorylation of the downstream mitogen-activated protein/extracellular signal-regulated kinase kinase (MEK) and extracellular signal-regulated kinase (ERK), resulting in malignant transformation and potential loss of differentiated functions [24]. Despite an excellent prognosis in the majority patients with PTC, about 20% of the cases develop regional recurrence or distant metastasis, and more than half will not respond to conventional therapy such as postoperative thyroid-stimulating hormone suppression and radioactive iodine (RAI) treatments [25]. Poor prognosis has been reported in these patients [26].

Targeted therapy, especially inhibition of BRAF^V600E^, may be an effective strategy for the treatment of metastatic PTC harboring this mutation. In fact, BRAF inhibitors have been already employed in the treatment of multiple cancers harboring the BRAF^V600E^ mutation. However, the therapeutic efficacies of these inhibitors vary from excellent responses in some cancers to drug resistance/tumor recurrence in others [27]. For instance, the therapeutic responses of BRAF-mutant cancers to these inhibitors ranged from a response rate of 48% in melanoma to 5% in colorectal cancer [28, 29]. In a phase I trial, treatment with the selective BRAF inhibitor vemurafenib in 3 patients with metastatic BRAF^V600E^-mutant PTC yielded a partial response in one and prolonged stabilization of disease in the others [30]. Several other Phase I and II studies using BRAF-inhibitors (dabrafenib and vemurafenib) have shown anti-tumor activity in a portion of patients with progressive BRAF^V600E^-mutant PTC [31–33]. However, small sample size and limited length of follow-up have made it difficult to predict which therapies are best suited for each patient, and which patients would likely best respond to the treatment.

Combination use of BRAF and MEK inhibitors has become a standard therapeutic approach in patients carrying a BRAF^V600E^ activating mutation. As for the observed effect of the combination therapy, BRAF and MEK inhibitors have shown significant improvements in clinical outcomes in BRAF^V600E^-mutant melanoma [34–40], non-small-cell lung cancer [41], and ATC [42]. However, little is known about the therapeutic effects of BRAF and MEK inhibitor combinations in advanced and metastatic BRAF^V600E^ mutation-harboring PTC. Also, there is as yet no way to clinically predict the therapeutic efficacies of BRAF-mutated cancers to BRAF and MEK inhibitors. Patient-derived organoids may provide a potential platform to test combination therapies aimed at finding drug synergisms and predicting therapeutic effect.

Previously, we have developed PTC organoids from human tumors and found that these models can accurately recapitulate the histological and genetic features of disease in vitro [43]. The robust organoid models derived from PTC tissues have potential to be used to aid in the selection of optimal anticancer drugs for individual patients. In our study, we established patient-derived PTC organoid models that recapitulate respective tumor characteristics. BRAF inhibitor monotherapy showed moderate sensitivity in treating BRAF^V600E^-mutant PTC organoids. The combination therapy of BRAF inhibitors with MEK inhibitors, receptor tyrosine kinases (RTK) inhibitors, or chemotherapeutic drugs significantly suppressed the growth of PTC organoids. Our study suggests that PTC organoids may be a potential preclinical tool used for the selection of drug treatment regimens for thyroid cancer patients.

## Methods

### Patient and sample collection

Papillary thyroid cancer tissues were gathered between August 2021 and June 2022 at Peking University Shenzhen Hospital, China. This study was approved by the Human Ethical Committee of the Hospital (Approval No. 2019-024), and written informed consent was signed prior to acquisition of samples from all patients involved. Patients’ characteristics are described in Table 1. Each tissue sample underwent histological assessment by two senior pathologists. The resected tumor tissues were placed into ice-cold Advanced DMEM/F12 medium (Cat. No. 12634-010, Gibco) and shipped to the laboratory on ice within 2 h of the surgery, for immediate further processing.

**Table 1:** Clinical characteristics of patients with papillary thyroid cancer

Sample No	Sex	Age (years)	Tumor size (cm)	BRAF mutation	Tumor stage	Node stage	Clinical stage
PTC-1	Female	60	1.0 × 0.7 × 0.3	+	T1	N0	I
PTC-2	Female	33	2.2 × 1.4 × 1.4	+	T2	N1a	I
PTC-3	Male	34	1.9 × 1.5 × 1.2	+	T1b	N1a	I
PTC-4	Male	33	3.0 × 2.4 × 1.6	+	T3	N0	I
PTC-5	Male	20	1.9 × 1.6 × 1.0	+	T1b	N1b	I
PTC-6	Female	28	3.5 × 2.3 × 1.8	−	T2	N1b	I
PTC-7	Female	31	2.5 × 2.0 × 2.0	−	T2	N0	I
PTC-8	Female	27	2.1 × 1.6 × 1.5	−	T1b	N1a	I
PTC-9	Male	32	3.5 × 3.0 × 3.0	−	T2	N1	I

### Organoid culture

Detailed procedures for PTC organoid derivation have been described previously by our group [43]. Briefly, the minced tumor tissues were digested with 5 mg/mL collagenase type II (Cat. No. 17101-015, Gibco) in the presence of Y-27632 dihydrochloride (10 µM, Cat. No. M1817, Abmole) for 40 min in a 37 ℃ shaking water bath. The digested tissues were further digested with 5 mL TrypLE Express (Cat. No. 12605-010, Gibco) for 5 min at 37 °C, strained over a 70 µm filter (Cat. No. 258368, NEST Biotechnology), and embedded in Matrigel (Cat. No. 356231, Corning). Tumor organoids were cultured in complete medium consisting of Advanced DMEM/F12 medium supplemented with 1% HEPES (Cat. No. 15630-080, Gibco), 1% GlutaMAX (Cat. No. 3505-0061, Gibco), 1% antibiotic–antimycotic (Cat. No. 15240-062, Gibco), 1 × B27 (Cat. No. 17504-044, Gibco), 1.25 mM N-acetyl-L-cysteine (Cat. No. A9165, Sigma-Aldrich), 10 mM Nicotinamide (Cat. No. N0636, Sigma-Aldrich), 500 ng/mL R-spondin-1 (Cat. No. 120-38, Peprotech), 100 ng/mL Noggin (Cat. No. 120-10C-1000, Peprotech), 5 ng/mL FGF-7 (Cat. No. 100-19, Peprotech), 10 ng/mL FGF-10 (Cat. No. 100-26, Peprotech), 50 ng/mL EGF (Cat. No. AF-100-15, Peprotech), 500 nM A83-01 (cat. no. SML0788, Sigma-Aldrich), and 10 μM SB202190 (Cat. No. S7067, Sigma-Aldrich). The medium was changed every 3–4 days. The organoids were visualized under a Carl Zeiss microscope (AXIO OBSERVER 3, Germany).

For passaging, growth medium was removed, and 3 mL of TrypLE Express was added to the Matrigel-cell suspension droplets and incubated at 37 °C for 3–5 min. Following mechanical blowing, AdDMEM/F12 containing 10% FBS would be added. Then, the suspension was centrifuged at 300 ×g for 5 min. PTC organoids were passaged at a 1:2–1:4 dilution every 1–4 weeks. For freezing, PTC organoids were resuspended in Recovery Cell Culture Freezing Medium (Cat. No. 12648-010, Gibco), cooled, and stored in liquid nitrogen. When required, organoids were thawed using standard thawing procedures, embedded in Matrigel and cultured as described above.

To compare the PTC organoid-forming efficiency, 1000 cells were seeded into a 24-well plate, and overlaid with organoid culture medium. Organoid numbers were counted with a light microscope after 10 days in culture.

### Histology and immunostaining

Tissues and organoids were fixed in 4% paraformaldehyde for 24 h, followed by dehydration, paraffin embedding, and serial sectioning at 4 μm. Tissues and organoids sections were subjected to hematoxylin and eosin (H&E) and immunofluorescence analysis using standard procedures. The histological diagnosis was made according to the standard classification. For immunofluorescence analysis, sections were boiled for 30 min in EDTA solution (pH 8.0) for antigen-retrieval, and blocked in 5% BSA blocking buffer for 30 min to reduce nonspecific staining. Primary antibodies against CK19 (1:200, Cat. No. Kit-0030, Maixin Biotech, China), galectin-3 (1:500, Cat. No. ab76245, Abcam), Ki-67 (1:200, cat. no. ab16667, Abcam), and BRAF (V600E mutant) (1:200, Cat. No. 29002, Cell Signaling Technology) were applied to the sections and incubated overnight at 4 °C. After being washed with PBS (3 × 10 min), the sections were subsequently incubated with secondary antibody, Cy3-labeled goat anti-mouse IgG (H + L) (1:300, Cat. No. A0521, Beyotime), Cy3-labeled goat anti-rabbit IgG (H + L) (1:300, cat. no. A0516, Beyotime, China), or Alexa Flour 488-labeled goat anti-mouse IgG (H + L) (1:300, Cat. No. A0428, Beyotime) for 1 h at room temperature. Nuclei were counterstained with DAPI (Cat. No. C1002, Beyotime) for 10 min and cover-slipped with an antifade polyvinylpyrrolidone mounting medium (Cat. No. 10981, Sigma-Aldrich). Finally, the images were captured on a Carl Zeiss microscope (Axio Imager.M2p, Germany).

### Whole-exome sequencing (WES) and data analysis

Genomic DNA from tumor tissues, tumor-derived organoids, and the paired tumor-adjacent normal tissues were isolated with an AllPrep DNA Mini Kit (Qiagen). The corresponding patients’ tumor-adjacent normal tissues were sequenced and used as a reference. Sequencing libraries were prepared using the Agilent Sure-Select Human All Exon kit (Agilent Technologies, CA, USA) according to the manufacturer’s protocol. The libraries were clustered using TruSeq PE Cluster Kit v4-cBot-HS (Illumina, San Diego, USA) on a cBot Cluster Generation System and sequenced using Illumina NovaSeq with 150 bp paired-end reads. The mean sequencing depth for paired tumor-adjacent normal tissues were approximately 100 ×(~ 10 Gb per sample), and for tumors and organoids were approximately 200 ×(~ 20 Gb per sample). Low-quality reads and adaptors were filtered using Fastp (v0.12.6) [44]. Single-nucleotide variants (SNVs) were detected using the Genome Analysis Toolkit (GATK, v4.1.9) [45]. Reads were aligned to the human reference genome GRCh37 with the Burrows-Wheeler Aligner (BWA, v0.7.17) [46]. SNVs and indels were analyzed by providing the reference (tumor-adjacent normal tissues) and tumors or organoids sequencing data to MuTect 2 and Strelka 2, respectively [47]. Effect predictions and annotations were added using ANNOVAR (version Feb 2016) [48]. To detect high quality somatic copy number variations (CNVs), BAM files were performed for read-depth variations using Control-FREEC v11.4 [49]. Mutational signatures were clustered with deconstructSigs [50], based on the set of 30 known mutation features.

### Drug treatment and organoid viability assay

PTC organoids were dissociated into single cells and small clusters, strained over a 70-µm filter to eliminate large organoids. Then organoids were resuspended in 2% Matrigel/growth medium (10,000 organoids/mL) and plated in Ultra Low Attachment Round Bottom 96-well plates (Cat. No. 7007, Corning) in triplicate. On the following day, drugs were diluted in organoid medium and added into each well with a six-point fivefold dilution series from 3.2 × 10^–3^ to 10 µM. For drug combination testing, PTC organoids were seeded into 96-well plates by following the same protocol as described above, and cultured with various doses of targeted agents, individually or in combination. The detailed information and maximum concentration of each drug is listed in Table 2.

**Table 2:** List of drugs used in this study

Target	Drug name	Company	Cat. No	Max screening conc. (µM)
BRAF^V600E^	Vemurafenib	TargetMol	T2382	10
BRAF^V600E^	Dabrafenib	MedChemExpress	HY-14660	10
MEK1	Selumetinib	MedChemExpress	HY-50706	10
MEK1/2	Trametinib	MedChemExpress	HY-10999	10
VEGFR, Raf-1	Sorafenib	MedChemExpress	HY-10201	10
VEGFR, PDGFRβ	Lenvatinib	MedChemExpress	HY-10981	10
VEGFR, c-Met	Cabozantinib	MedChemExpress	HY-13016	10
VEGFR, EGFR	Vandetanib	MedChemExpress	HY-10260	10
VEGFR, PDGFRβ	Sunitinib	MedChemExpress	HY-10255A	10
DNA topoisomerase II	Doxorubicin	MedChemExpress	HY-15142A	10
Microtubule	Vincristine	MedChemExpress	HY-N0488A	10
Microtubule	Paclitaxel	TargetMol	T0968	10
DNA synthesis	Cisplatin	TargetMol	T1564	10

Organoid viability was analyzed using the CellTiter-Glo^®^ 3D Reagent (Cat. No. G9683, Promega) according to manufacturer’s specifications following 5 days of drug incubation, and results were normalized to dimethyl sulfoxide (DMSO)-treated control organoids. For testing the combined effect of two drugs, organoids were treated with each drug alone or in combination before undergoing a viability assay. Luminescence reading was performed in a Synergy H1 Hybrid Multi-Mode Microplate Reader (BioTek, USA). All experiments were performed in duplicate in three biological replicates (different passages of PTC organoids). Luminescence readings from drug-treated wells were normalized against that of DMSO-treated control wells, and drug sensitivity was shown by the half-maximal inhibitory concentration (IC_50_), the slope of the dose–response curve, and the area under the dose–response curve (AUC). For PTC organoids that were completely resistant to a drug, the values of the IC_50_ and AUC could not be generated, but for analysis purposes, they were given the maximum IC_50_ of the drug or AUC = 1 within the organoid panel.

### Statistical analysis

Results were expressed as mean ± SEM and differences among groups were performed by one-way ANOVA followed by Tukey multiple comparison test using the SPSS 19.0 software (SPSS, Inc., IL, USA). A p-value < 0.05 was considered significant. All statistical analyses were performed using the GraphPad Prism software version 7.0 (GraphPad Software, Inc., CA, USA) or SPSS 19.0. Drug interactions were statistical analyzed with ComboSyn Software (ComboSyn Inc. Paramus, NJ) according to the Chou-Talalay method [51, 52].

## Results

### Establishment of human PTC-derived organoid lines

**Fig. 1:**
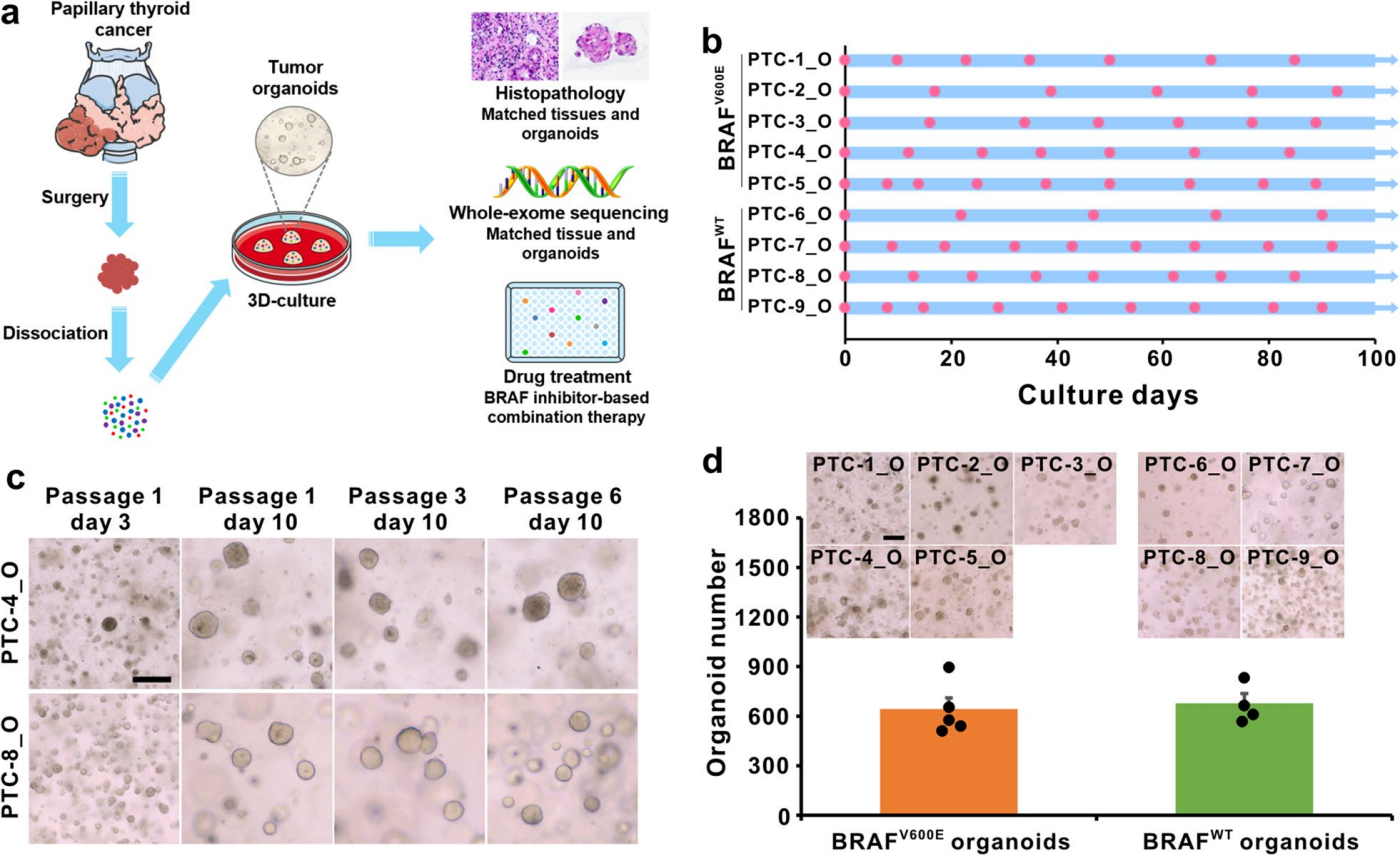
Establishment of patient-derived PTC organoids harboring BRAF^V600E^ mutation or wild-type (WT). **a** Overview of the procedure. Nine PTC organoids were derived and analyzed by histological characterization, DNA-sequencing, and drug sensitivity assays. **b** Expansion potential of nine PTC organoid cultures. Dots on the graph represent passage, arrows represent continuous expansion. PTC, papillary thyroid cancer; O, organoid. **c** Representative images of long-term cultured PTC organoids. Organoid cultures were derived from PTC-4_O and PTC-8_O. Scale bar, 100 µm. **d** Organoid formation efficiency of BRAF^V600E^ PTC organoids and BRAF^WT^ PTC organoids. Data represent the mean ± SEM of organoid number in PTC organoid lines. Scale bar, 100 µm

Each fresh surgically resected PTC tissue from each patient was cut into several pieces that were processed for organoid culture, histological analysis, DNA-sequencing, and drug sensitivity analyses (Fig. 1a). Based on our previously reported three-dimensional (3D) culturing conditions of long-term expansion of PTC organoids [43], we dissociated tumor tissues into single cells and cell clumps through mechanical disruption and enzymatic digestion, embedded into Matrigel and submerged in organoid culture medium. Tumor cells and cell pellets generally formed round organoids within 1–2 weeks. These independent PTC organoids derived from 9 different patients have been propagated by serial passaging and successfully cryopreserved and recovered as two-dimensional (2D) cell lines. PTC organoid growth rates showed significant variability between patients, with passaging intervals varying from 1 to 3 weeks and split ratios ranging from 1:2 to 1:4. The growth and passage of these PTC organoids were recorded (Fig. 1b). As representative images shown in Fig. 1c, all established PTC organoids can be cultured and passaged more than 3 months without showing any decline in growth rate and significant change in morphology. The clinicopathological diagnosis revealed that PTC-1 to PTC-5 were BRAF^V600E^ mutant-type, and PTC-6 to PTC-9 were BRAF wild-type (Table 1). Immunofluorescence staining and WES showed that the established PTC organoids also captured the BRAF^V600E^ mutation of the primary tumors (Figs. 3, 4). There were no obvious differences in the organoid-forming efficiency between wild-type and BRAF^V600E^-mutant organoids (Fig. 1d), indicating that the BRAF^V600E^ mutation does not improve PTC organoid self-renewal in vitro.

### PTC organoids recapitulate the original histological characteristics

**Fig. 2:**
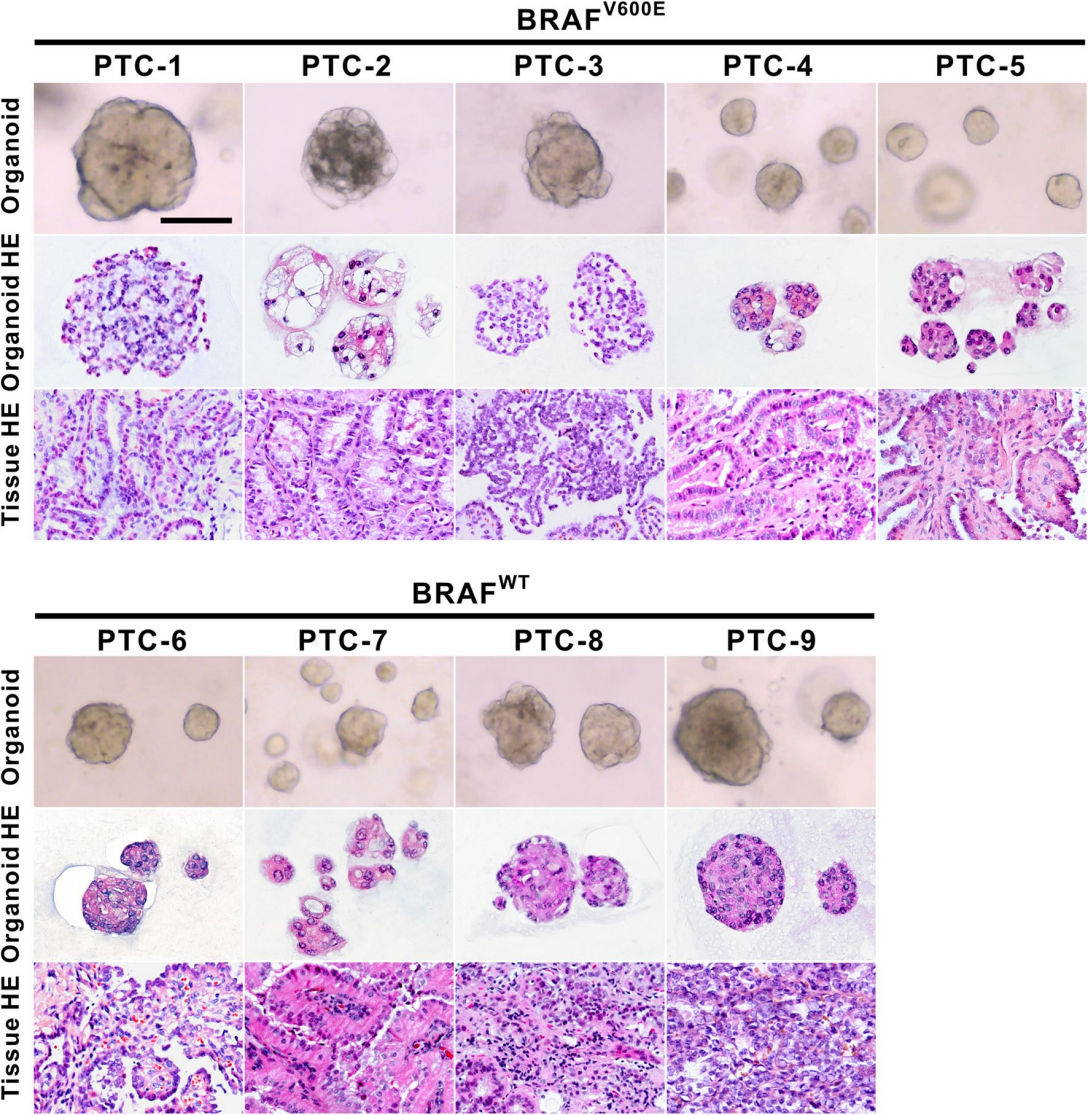
Histopathological Characteristics of PTC organoids with BRAF^V600E^ mutation or wild-type and their parental tumors. Representative brightfield images of PTC organoids (top), and H&E staining of organoids (middle) and tumor tissues (bottom). Passage numbers of PTC organoid lines were: PTC-1_O, P3; PTC-2_O, P3; PTC-3_O, P2; PTC-4_O, P4; PTC-5_O, P3; PTC-6_O, P2; PTC-7_O, P4; PTC-8_O, P3; PTC-9_O, P4. PTC, papillary thyroid cancer; WT, wild-type. Scale bar, 100 µm

To compare the morphological and histological features of PTC organoids with their corresponding parental tumors, we performed H&E staining and immunofluorescence analysis. As shown in Fig. 2, PTC organoids showed similar histological patterns to those of the originating tumor tissues. PTC organoids derived from different patients displayed distinct morphological and histological features, as based on brightfield microscopy and H&E staining. PTC organoids grew either as dense structures or as cystic structures. For example, we observed that PTC-2_O exhibited a multi-hole cystic structure, whereas PTC-4_O appeared as solid sphere-like shape (Fig. 2).

**Fig. 3:**
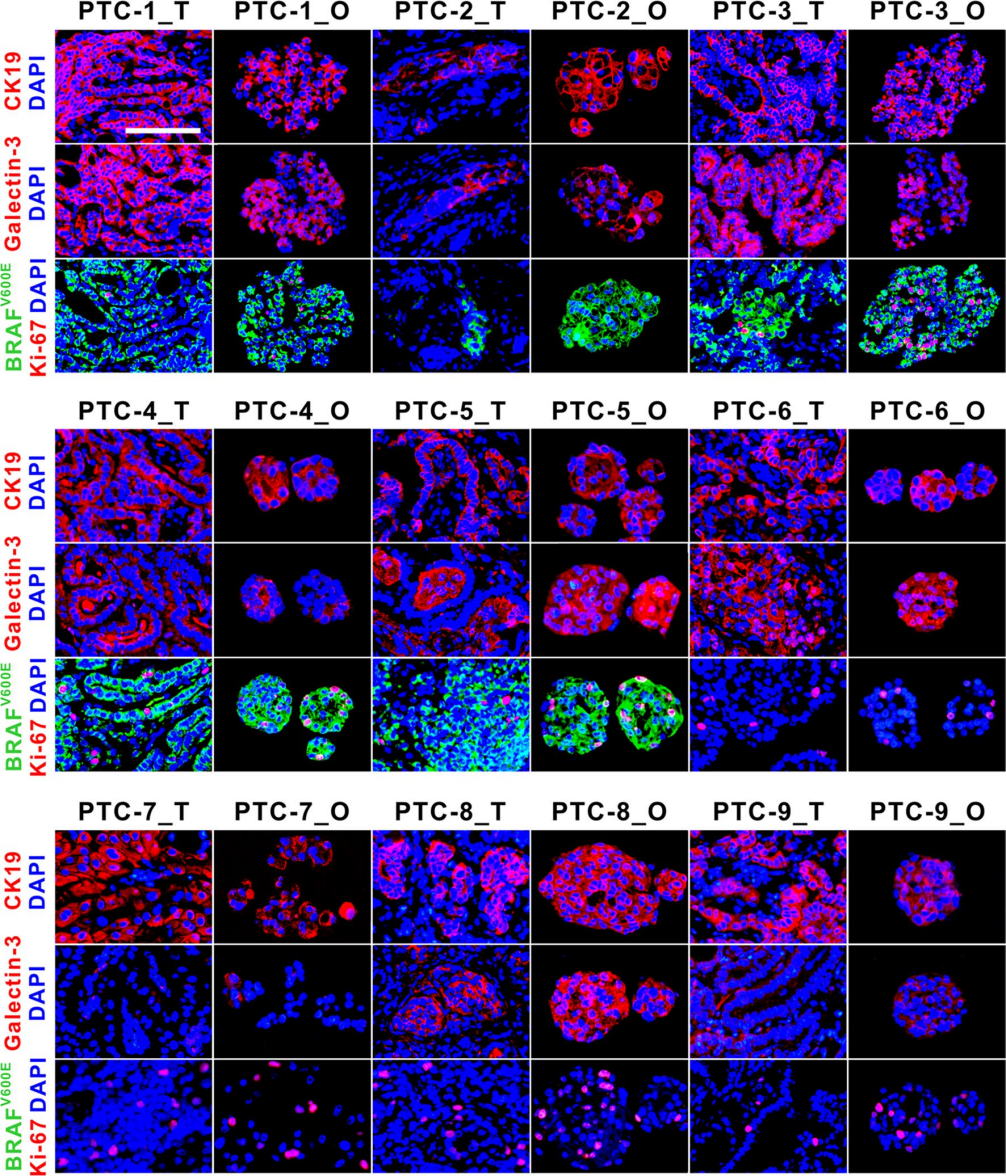
Immunofluorescence staining of CK19, galectin-3, BRAF^V600E^, and Ki-67 on PTC organoids and the parental tumors. Passage numbers of PTC organoid lines were: PTC-1_O, P3; PTC-2_O, P3; PTC-3_O, P2; PTC-4_O, P4; PTC-5_O, P3; PTC-6_O, P2; PTC-7_O, P4; PTC-8_O, P3; PTC-9_O, P4. PTC, papillary thyroid cancer. T, parental tumor; O, organoid. Scale bar, 100 µm

Next, we performed the immunofluorescence analyses using a panel of antibodies against tumor subtype markers. We observed CK19 and galectin-3, two clinical and laboratory markers for the detection of PTC, to be expressed in all PTC organoids and the matched tumors (Fig. 3). However, differences could be seen in the expression levels of the two markers among these patients. For BRAF^V600E^ expression status, PTC-1_T to PTC-5_T exhibited a mutant staining pattern, as was recapitulated in the derived organoids. Complete absence of BRAF^V600E^ expression pattern was observed in PTC-6_T to PTC-9_T, and their corresponding organoid lines. These immunostaining results corresponded to the patient pathology reports (Table 1). Ki-67, a proliferation marker, was present in almost all the tumors and the derived organoids (Fig. 3). However, the expression of Ki-67 in the BRAF^V600E^-mutant PTC organoids showed no obvious differences with BRAF wild-type organoids. The expression of Ki-67 in some PTC organoids (PTC-3_O and PTC-9_O, for instance) appeared to be increased compared to that in primary tumor tissue. PTC tissues, however, contain stromal cell and immune cell types, which could explain the difference. Furthermore, Ki-67 staining revealed that higher cell proliferation in parental tumors could be propagated as their organoids. Collectively, these results indicate that PTC organoids clearly reflect the histological features and marker expressions of their parental tumors.

### PTC organoids preserve the mutational landscape of the corresponding tumors

**Fig. 4:**
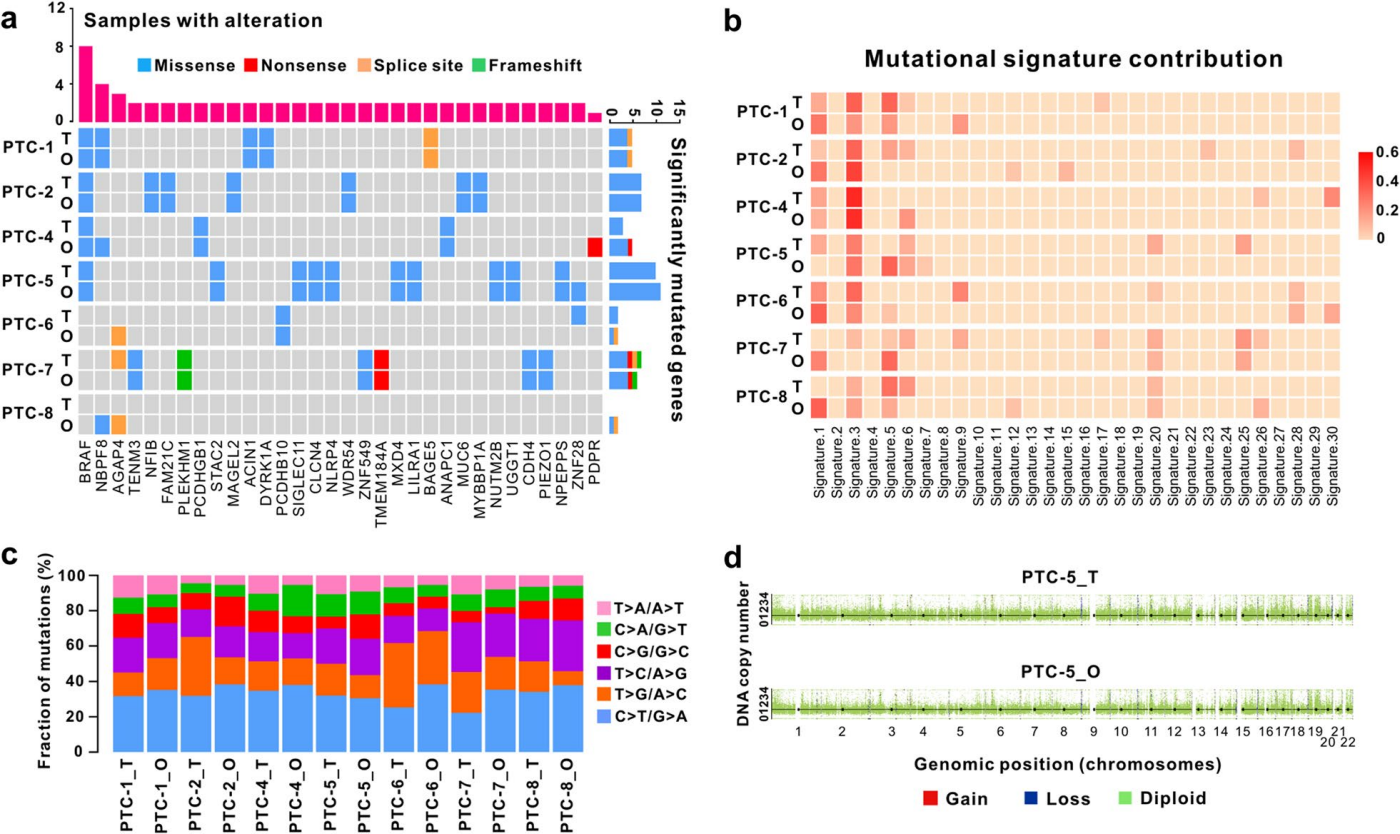
Genetic characterization of PTC tissues and the derived organoids. **a** Heatmap analysis of the somatic mutated cancer genes in PTC organoids with those in parental tumors. **b** Heatmap displaying the mutational signature contributions of organoids and respective tumors. **c** Histogram showing the proportions of base substitutions in the paired tumor tissues and organoids. **d** Representative gene copy number variations of the paired PTC organoids and tumor tissues. Passage numbers of PTC organoid lines were: PTC-1_O, P3; PTC-2_O, P4; PTC-4_O, P4; PTC-5_O, P5; PTC-6_O, P3; PTC-7_O, P5; PTC-8_O, P4

To determine whether the PTC organoids maintain the mutational landscape present in their originating tumors, we performed WES on 7 organoid lines and their corresponding tumor tissues. There was no sufficient tumor tissue available from PTC-3 and PTC-9 for DNA-sequencing. We filtered variants and excluded polymorphisms present in organoids and tumors by comparing them to the analysis of paired patients’ tumor-adjacent normal tissues. The PTC organoids showed a heterogeneous set of cancer driver genes affected by missense, nonsense, splice site, or frameshift mutations, some of which displayed a variable pattern of alteration (Fig. 4a). Comparative analysis showed that the mutation spectrum present in the primary tumor tissues were highly maintained in the derived PTC organoids (Fig. 4a). Importantly, the PTC organoid lines recapitulated the majority of the most frequently mutated genes in PTC patients. For example, somatic mutations in *BRAF*^*V600E*^, the most frequently mutated gene in PTCs, was mutated in 5 of 9 patients.

We analyzed somatic base substitutions in both organoids and the paired tumors and plotted the relative mutational-signature contributions of each sample in 30 mutational signatures. The proportion of base substitutions and contribution of signatures were well maintained among tumors and the paired organoids (Fig. 4b, c). The most frequent base substitutions in PTC tumor tissues and organoids were C > T/G > A transitions (Fig. 4c), which agrees to what has been previously reported [43]. CNVs analysis demonstrated similar DNA copy number gain and loss patterns in PTC tissues and the corresponding organoids (Fig. 4d, Additional file 1: Fig. S1). Like many other cancers, thyroid cancers also have intratumoral heterogeneity [53, 54]. In our study, tumor tissue used to generate organoids come from different tumor sites compared to the parental tumor used for WES. Although we tried to develop organoids from tumor sites close to the parental tumor, some differences in mutational spectrum (mutational signature 1, for instance) between organoids and their parental tumor may still occur. This is probably due to the intratumor mutational heterogeneity. Overall, we demonstrate that PTC organoid lines recapitulate the mutational landscape of the parent tumors.

### Individual patient-derived PTC organoids show specific sensitivity to drug therapy

**Fig. 5:**
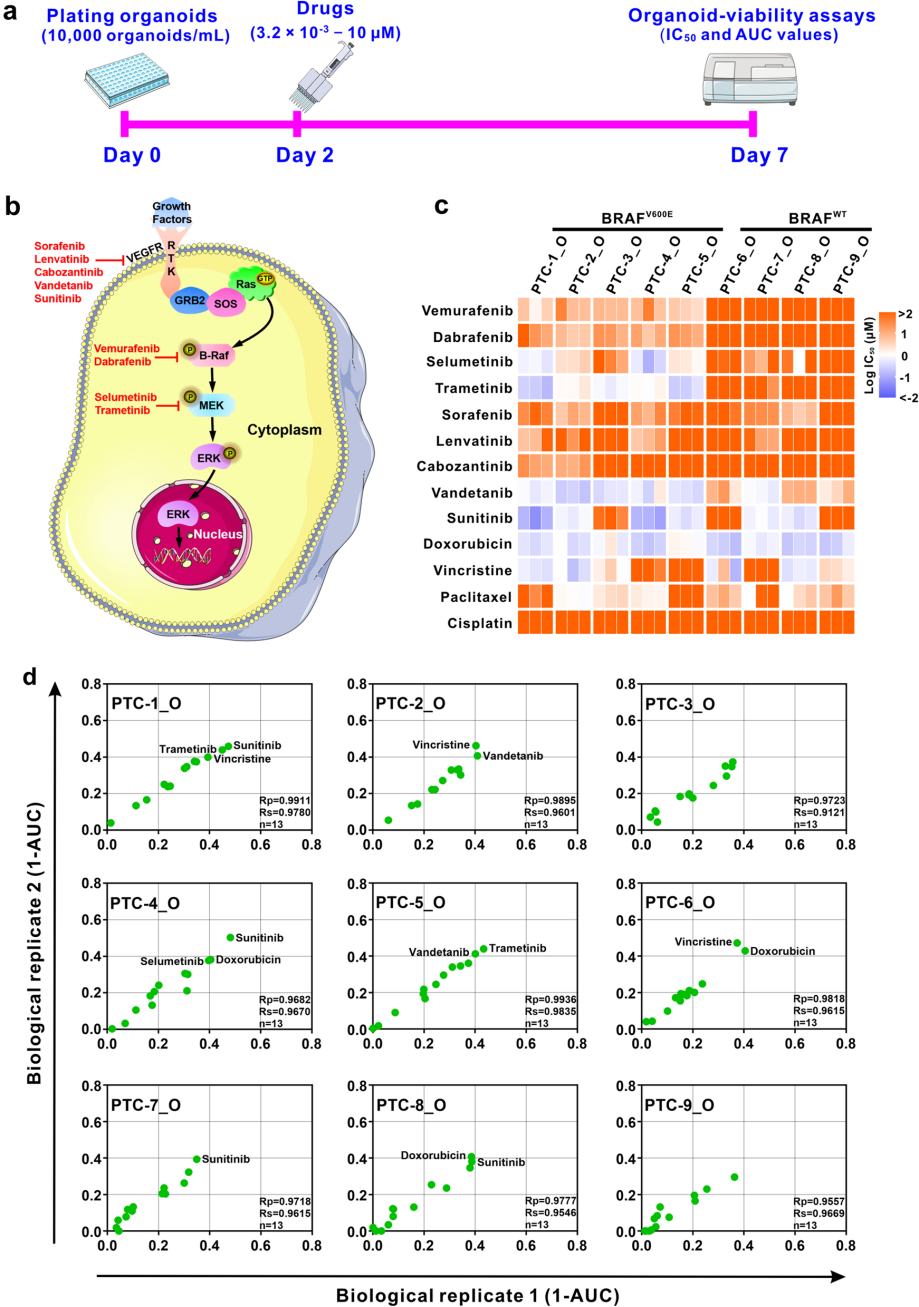
PTC-derived organoids as a platform for predicting drug responses. **a** Protocol for the treatment of PTC organoids with various anticancer drugs. Organoids were plated (10,000 organoids/mL) and cultured for 2 days, and drugs were diluted in organoid medium and added into each well with six-point fivefold dilution series (3.2 × 10^–3^ µM to 10 µM). After 5 days of treatment with the drugs, organoid viability was examined by using the CellTiter-Glo® 3D Reagent. **b** Simplified scheme of the RAS/RAF/MEK/ERK signaling pathway including targeted drugs used. **c** Heatmap of logIC_50_ values for 13 anticancer drugs used to treat 9 PTC organoid lines by applying nonlinear regression. The corresponding colors for logIC_50_ are depicted in the legend. Orange indicates high IC_50_ values, violet indicates low IC_50_ values. The assay was performed with 3 biological replicates (different passages of PTC organoids). **d** Representative scatterplots of 1-AUC values generated from two biological replicates of the drug screening data. Each data point represents 1-AUC for a drug used to treat the indicated PTC organoids. Passage numbers of PTC organoid lines were: PTC-1_O, P3, P4 and P5; PTC-2_O, P2, P3 and P4; PTC-3_O, P3, P4 and P5; PTC-4_O, P3, P4 and P5; PTC-5_O, P3, P4 and P5; PTC-6_O, P3, P4 and P5; PTC-7_O, P4, P5 and P6; PTC-8_O, P3, P4 and P5; PTC-9_O, P4, P5 and P6

**Fig. 6:**
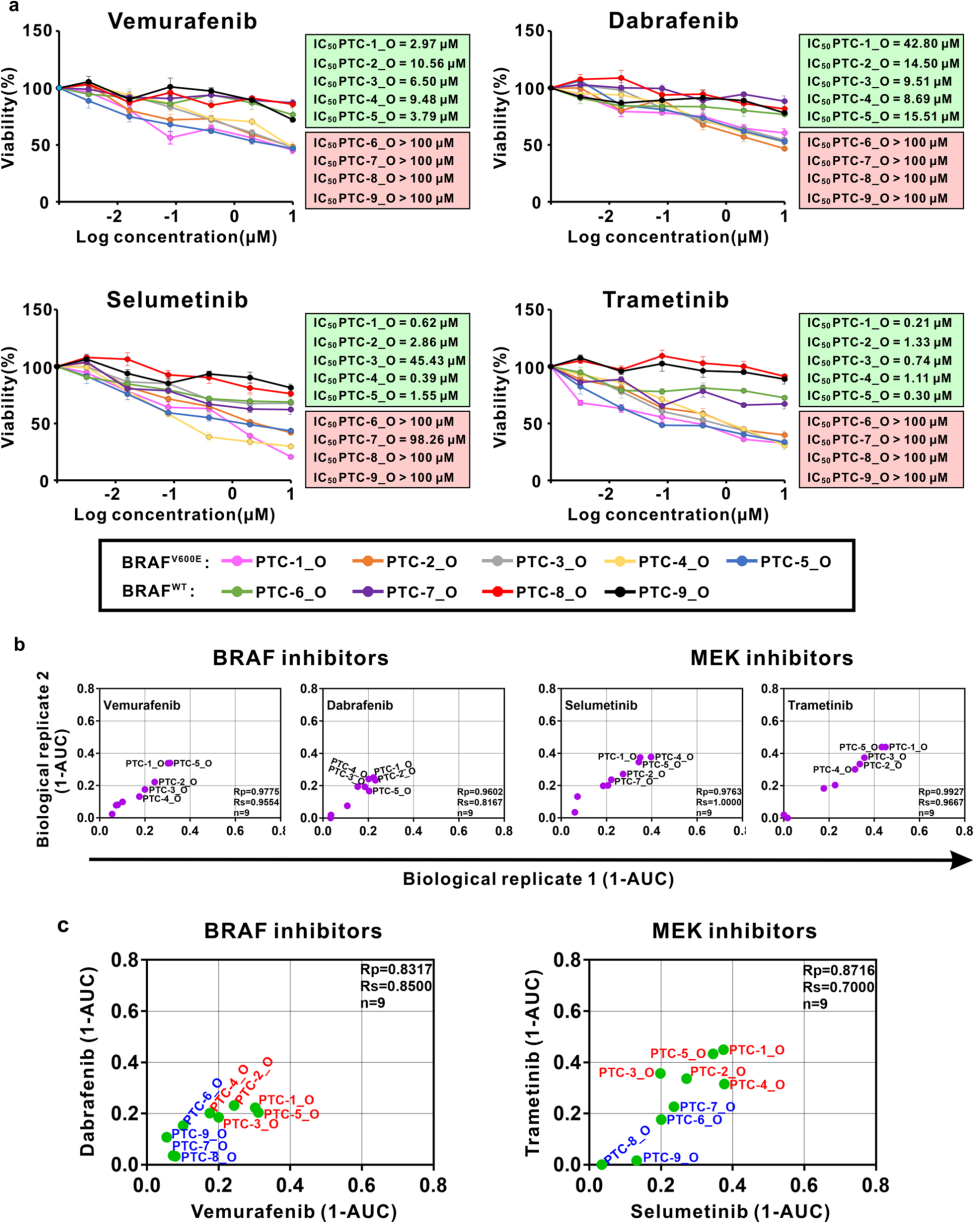
Sensitivity of PTC-derived organoid lines for BRAF and MEK inhibitors. **a** Dose–response curves after 5 days of treatment with BRAF and MEK inhibitors. Each data point represents mean ± SEM of 3 independent biological replicates. IC_50_ values are calculated and indicated beside the curve graphs. **b** Scatterplots of the correlation of 1-AUC values for targeted agents and chemotherapeutic drugs screened by two biological replicates (different passages of PTC organoids). Each data point represents 1-AUC for a PTC organoid line treated by the indicated drug. **c** Drugs with a common target have similar activity profiles across the PTC organoid lines. 1-AUC values are plotted for the inhibitors of BRAF^V600E^ (vemurafenib and dabrafenib) and MEK (selumetinib and trametinib)

To explore the potential of PTC organoids as preclinical in vitro disease models for the evaluation of drug response, we conducted dose titration assays to examine the effects of 13 anticancer drugs on 9 established organoid lines. PTC organoids were dissociated into single cells and small clusters, suspended in organoid medium containing 2% Matrigel, and dispensed into 96-well plates. Two days after plating, a dilution series of drugs were added, and organoid viability was measured 5 days after supplementing the drugs (Fig. 5a). Organoid viability assays were normalized to the DMSO-treated control organoids to ensure that toxicity was specific to the drug effects. Drugs were chosen based on their clinical use in thyroid cancer therapy, and included investigational agents being tested in clinical trials such as drugs targeting the RAS/RAF/MEK/ERK pathway (Fig. 5b). We were able to generate reproducible dose–response curves for drugs and calculated IC_50_ and AUC through dose titration assays. All experiments were performed in duplicate in three biological replicates (different passages of PTC organoids) and replicate AUC values were highly correlated (Pearson correlation, Rp > 0.95) (Fig. 5d). Moreover, all the drugs were screened at least twice on separate plates and good correlations between the experimentally determined values of AUC were observed (Fig. 6b, Additional file 2: Fig. S2).

Organoid viability assays revealed divergent sensitivities and varying IC_50_ values to drug treatments among the PTC organoid lines (Figs. 5c, 6a, Additional file 3: Fig. S3), indicating patient-specific drug responses. At the same time, drug-response assays demonstrated distinct responses of different drugs on individual PTC organoid lines (Fig. 5c, d), indicating drug-specific sensitivities. BRAF^V600E^ inhibitors, vemurafenib and dabrafenib, showed different activities depending on the patient’s tumor, and in particular, on the BRAF mutation status. BRAF^V600E^-mutant organoids from patient 1 to 5 displayed modest sensitivities to vemurafenib and dabrafenib, whereas BRAF wild-type organoid lines demonstrated no responses (Figs. 5c, 6a, b), suggesting a correlation between drug sensitivities and their mutational profiles in organoids. As predicted, most BRAF^V600E^-mutant organoids are sensitive to drugs targeting the MEK signaling pathway, whereas BRAF wild-type organoids are resistant (Fig. 5c, 6a, b). Furthermore, we observed drugs that inhibit the BRAF^V600E^ and MEK1/2 signaling pathways had comparable activity across the organoid lines. As shown in Fig. 6c, a similar sensitivity pattern was observed for the BRAF^V600E^ inhibitors vemurafenib and dabrafenib (Pearson correlation, Rp = 0.83), and the MEK inhibitors selumetinib and trametinib (Pearson correlation, Rp = 0.87).

Tyrosine kinase inhibitors (TKIs), such as lenvatinib and sorafenib, have been approved by the US Food and Drug Administration (FDA) for the treatment of RAI-refractory metastatic DTC. Of the five TKIs tested, vandetanib showed the most significant anticancer effect, and sunitinib showed an inhibitory effect on 6 of 9 PTC organoid lines (Fig. 5c, Additional file 2: Fig. S2, Additional file 3: Fig. S3). The other 3 drugs, sorafenib, lenvatinib and cabozantinib, only exhibited weak inhibitory effects on the PTC organoids. Conventional chemotherapeutic drugs, such as microtubule-targeting regimens vincristine and paclitaxel, and topoisomerase-targeting agent doxorubicin, induced cell death in various PTC organoids with different sensitivities. For example, doxorubicin resulted in a growth-inhibitory effect on all the 9 PTC organoids, whereas vincristine suppressed formation in 6 of 9 PTC organoids (Fig. 5c, Additional file 2: Fig. S2, Additional file 3: Fig. S3). Interestingly, all the 9 PTC organoid lines were relatively resistant to cisplatin, a platinum-containing DNA crosslinker (Fig. 5c, Additional file 2: Fig. S2, Additional file 3: Fig. S3). Overall, these experiments showed that the responses of PTC organoids derived from different patients to various drug treatments are heterogeneous. These results demonstrated that PTC organoids can serve as a predictive model for preclinical evaluation of personalized drug treatment responses.

### PTC organoids harboring BRAF^V600E^ mutation exhibited increased sensitivity to BRAF inhibitor-based combination therapies

**Fig. 7:**
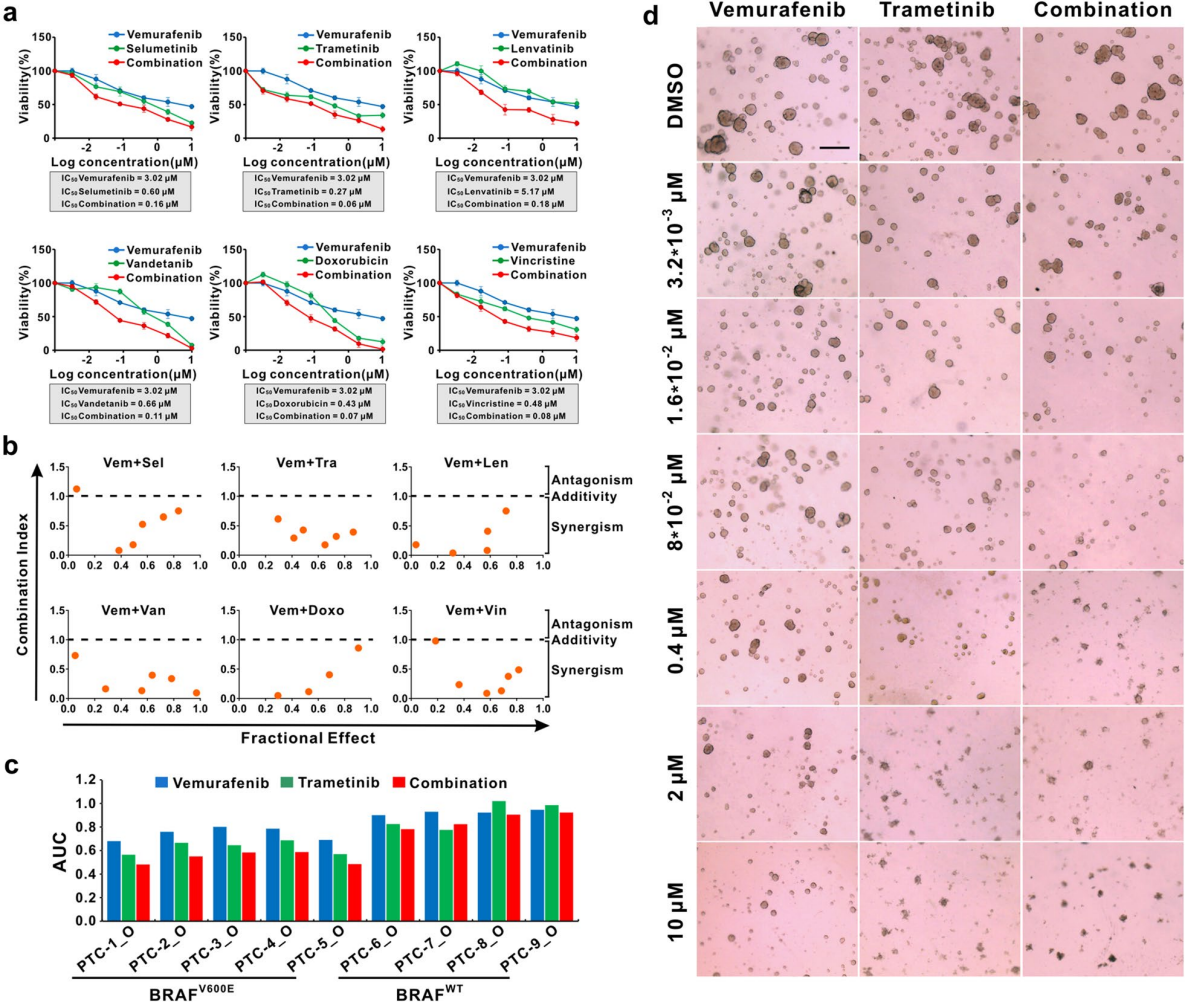
Combination of BRAF inhibitor with MEK inhibitors, RTK inhibitors, or chemotherapy drugs in PTC organoid lines. **a** Combination drug test of vemurafenib with selumetinib, trametinib, lenvatinib, vandetanib, doxorubicin, and vincristine in PTC-1 organoid line. Each data point represents mean ± SEM of 3 independent biological replicates. Organoid viability was detected by CellTiter-Glo assay after 5 days of drug treatment, and results were calculated relative to DMSO-treated control organoids. **b** The Combination Index (CI) versus Fractional Effect plot for the indicated drug combinations, according to the Chou-Talalay dose–effect method. The CI < 1.0, CI = 1.0, and CI > 1.0 indicate synergism, additivity, and antagonism, respectively. **c** Response of each PTC organoid line to vemurafenib, trametinib, or combination therapy. AUC generated from biological replicates of a dose–response curve to drug alone and in combination. **d** Representative bright-field images of vemurafenib and trametinib-treated PTC organoids (PTC-1_O), alone or in combination. Scale bar, 100 µm

Besides surgical intervention, drug therapy as monotherapy or in combination is an alternative treatment option for locally advanced or metastatic thyroid cancer. However, the exact benefit of combining these drugs has remained questionable. We explored BRAF inhibitor-based combination therapies in our established BRAF^V600E^-mutant PTC organoid lines. The Combination Index (CI) values of the indicated drug combinations were analyzed using the ComboSyn software. The CI values < 1.0 indicate synergism, whereas CI values > 1.0 indicate antagonism of the combination treatment. Treatment of PTC organoids with vemurafenib showed decreased cell viability; however, the combination of vemurafenib with a MEK inhibitor, RTK inhibitor or chemotherapy drug exhibited a synergistically better inhibitory effect on PTC-1_O as compared to either monotherapy (Fig. 7a, b). In the other 4 patients with the BRAF^V600E^ mutation (PTC-2 ~ PTC-5), their organoid lines also responded better to the combination therapy than either single agent (Additional file 4: Fig. S4, Additional file 5: Fig. S5).

On the basis of previous cell line studies and clinical trials showing efficacy of the combination of BRAF inhibitors with MEK inhibitors in BRAF^V600E^-mutant human cancers, we examined the sensitivity of BRAF^V600E^-mutated PTC organoids with both single and double agent treatment of vemurafenib and trametinib. In the in vitro assays, vemurafenib and trametinib reduced PTC organoid growth in a dose-dependent manner, individually or in combination (Fig. 7a, d). Vemurafenib alone had only a modest impact on cell viability in BRAF^V600E^-mutant PTC organoids, but the combination of vemurafenib and trametinib induced a greater effect in these same organoid cultures (Fig. 7c, d). The four wild-type PTC organoids were insensitive to single and combination therapy drugs (Fig. 7c). Our data indicates that the BRAF and MEK inhibitor combinations may be an excellent clinical option to achieve a synergistic killing effect for PTC. The combination of BRAF inhibitors with MEK inhibitors, RTK inhibitors or chemotherapy drugs can serve as a potential treatment strategy to enhance the therapeutic efficacy in PTC harboring the BRAF^V600E^ mutation.

## Discussion

Precision medicine is a promising strategy for providing the right therapy for the right patient, which is much required for cancer patients. The lack of a reliable method to predict treatment response is one of the major limitations in clinical oncology. In the past few years, organoid methodology has gained a lot of attention for modeling healthy and malignant tissues. Organoids hold great promise for precision medicine, mirroring its value in basic, translational, and clinical cancer research [55]. Tumor organoids have been proven to more faithfully recapitulate the histological architectures, molecular characteristics, genomic profiles, mutational signatures, and expression features of primary tumors. In our study, we established BRAF^V600E^-mutant and wild-type PTC organoid models in vitro. Under the optimized culture condition, PTC organoids were cultured and passaged for more than 3 months without showing any decrease in growth rate and significant change in morphology. The histopathological characteristics, molecular hallmarks, genetic profiles, and mutational signatures of PTC organoids were concordant with those detected in the matched patient tumors. More importantly, the BRAF^V600E^ mutation status present in parent tumors were well conserved in the derived organoids.

During the past decades, a great number of anticancer drugs developed from screening 2D cell lines have failed in in vivo studies and clinical trials. PDOs open a door to fill the gap between drug testing on cell lines and clinical trials. Researchers have presented a range of examples for personalized medicine applying organoids in various cancer types [7, 8, 56, 57]. In the current study, anticancer drug screening using PTC organoid cultures indicated remarkable differential responses of different patients to the treatment. The fact that a variable response to targeted agents and chemotherapeutic drugs was observed in vitro indicates that this method records intra- and interindividual differences. Therefore, by predicting patients’ responses to drugs using their tumor organoids as ‘‘proxies’’, the best treatment strategy for the individual patients may be selected.

BRAF^V600E^ mutation occurs in most PTCs and some ATCs deriving from PTCs, and it is recognized as the most frequently genetic alteration occurring in thyroid cancers [22, 58, 59]. This make BRAF^V600E^ an underlying prognostic biomarker and therapeutic target for thyroid cancer. MEK inhibitor, targeting the RAS/RAF/MEK/ERK signaling pathway, has also been shown separately to enhance survival in patients with metastatic melanoma [60]. Treatment of BRAF-mutant PTC cell lines with BRAF inhibitor decreased phosphorylation of MEK and subsequently ERK1/2, blocked cell cycle progression, and inhibited tumor xenograft growth [61]. In our study, we identified BRAF and MEK inhibitions were efficacious only in BRAF^V600E^-mutant organoids, but not in BRAF wild-type organoids, suggesting that PTC organoids may be helpful for predicting patients’ response to targeted therapy. With the introduction of BRAF and MEK molecular targeted therapies for patients with advanced or metastatic PTC, determination of BRAF mutation status is crucial to selecting patients who will most likely benefit from this therapy.

Although BRAF^V600E^ is considered a promising therapeutic target for several cancers, especially for BRAF mutation-harboring melanoma [28, 62], the BRAF inhibitor monotherapy was less effective than anticipated in clinical trials of patients with PTC harboring the BRAF^V600E^ mutation [32, 33]. In our study, BRAF^V600E^-mutant PTC organoids were only moderately sensitive to the BRAF^V600E^ inhibitor (vemurafenib or dabrafenib) treatment. To prevent drug resistance and/or improve response rate, combination therapy targeting BRAF and MEK was assessed and demonstrated synergistic benefit. Many studies have verified that the addition of a MEK inhibitor to a BRAF inhibitor improved progression-free survival and overall survival over BRAF inhibitor monotherapy in patients with BRAF^V600E^-mutant melanoma [34–40]. In an open-label Phase II trial, dabrafenib plus trametinib therapy for 16 patients with BRAF^V600E^-mutant ATC also generated a significantly higher response rate [42]. Dual treatment with dabrafenib and trametinib effectively killed BCPAP cells (BRAF^V600E^-harboring thyroid cells) after 2 days, indicating that these cells were sensitive to the drugs [63]. Given the success of the combined treatment in melanoma, continuing efforts are recommended in PTC, which can now be explored with the organoid models in our present study. The combinations of BRAF inhibitor with MEK inhibitor resulted in enhanced treatment response compared with BRAF inhibitor monotherapy. The promising results from PDOs may provide the basis for clinical treatment of locally advanced or metastatic BRAF^V600E^-mutant PTC patients.

Our study identified a dozen BRAF inhibitor-based drug combination strategies with good synergistic effects against PTC organoids. The TKIs are a class of small molecules or peptides that have been developed and clinically tested that inhibit either cytosolic or receptor tyrosine kinases [64]. These enzymes can phosphorylate many regulatory proteins in the cell, and can trigger signal transduction cascades that regulate many cellular functions such as proliferation, differentiation, and metabolism [65]. We tested the PTC organoids’ sensitivities to several commonly used TKIs, and these assays revealed differential drug responses of individual PTC organoid lines. Interestingly, vandetanib and sunitinib showed more inhibitory effect on PTC organoid lines than sorafenib, lenvatinib and cabozantinib, indicating the two TKIs may be especially beneficial for PTC patients.

BRAF inhibition introduced by V600E mutation causes a rapid feedback activation of human epidermal growth factor receptor (HER)/epidermal growth factor receptor (EGFR) in colorectal cancer cell lines [66–68]. Actually, inhibition of the BRAFV600E/MEK/ERK axis in thyroid cancer cells also results in reactivation of a variety of RTKs such as HER2/HER3 [69, 70], platelet-derived growth factor receptor-beta (PDGFRβ) [69], and EGFR [71]. Treating thyroid cancer cells with BRAF inhibitors will set free C-terminal binding proteins (CTBPs), which was revealed as important transcription factors to promoting expression of HER3 [69]. The reactivation of HER family members (HER2/HER3) induced the relieving of MAPK/ERK pathways inhibition, leading to the resistance towards BRAF inhibitors in thyroid cancer cells [69, 70]. Further study demonstrated that combination of HER inhibitor to BRAF/MEK inhibitor overcomes resistance to vemurafenib in BRAF mutated thyroid cancer cells [69]. In addition to the activation of HER family members, there was upregulation of PDGFRβ in response to vemurafenib in thyroid cancer cell lines [69]. This is of interest because activation of PDGFRβ has been proposed as a mechanism of acquired resistance to vemurafenib in patients with metastatic melanoma [72]. Furthermore, Notarangelo and colleagues reported that the exposure of thyroid cancer cells to vemurafenib resulted in a rapid feedback activation of EGFR pathway, and dual EGFR and BRAF blockade induces suppression of ERK signaling, inhibition of cell proliferation, and synthetic lethality [71]. In our study, BRAF^V600E^-mutant PTC organoids derived from some patients were sensitive to vandetanib (inhibits EGFR, vascular endothelial growth factor receptor (VEGFR), and rearranged during transfection (RET)) and/or sunitinib (inhibits PDGFRβ, VEGFR, and fibroblast growth factor receptor (FGFR)). The combination of vandetanib/sunitinib with BRAF inhibitor exhibited more inhibitory effect on PTC organoids than BRAF inhibitor alone. Feedback mechanism that upregulation of EGFR/PDGFRβ which in turn results in reactivation of MAPK pathway may be a probable explanation for drug resistance in BRAF inhibitor monotherapy. Our study provides basis for in vivo and clinical studies using combination of BRAF inhibitor and vandetanib/sunitinib may become a potential therapeutic regimen for BRAF^V600E^-positive patients.

## Conclusions

In summary, we established BRAF^V600E^-mutant and wild-type PTC organoid models, and described histopathological and genomic characteristics, as well as their responses to anticancer drugs, alone or in combination. PTC organoids may help to predict drug response for individual patients and identify which patients are most likely to benefit from BRAF-oriented therapy. We elucidated that organoid model can be used as an ex vivo platform for evaluating drug sensitivity and finding combination regimen aimed at enhancing therapeutic effect. The combination of BRAF inhibitor with MEK inhibitor to enhance the therapeutic efficacy deserves further investigating in more organoids derived from patients and in PDX models. We actually tried to apply PTC organoids to establish PDX models for in vivo drug testing. Unfortunately, these PTC organoids were unable to form tumors in NSG mice. Additional studies on clinical practices are also necessary to evaluate the efficacy and safety of BRAF-based combination therapies.

## Supplementary Information

**Additional file 1: Figure S1.** Gene copy number variations of paired PTC organoids and tumor tissues.

**Additional file 2: Figure S2.** Scatterplots of the correlation of 1-AUC values for targeted agents and chemotherapeutic drugs screened by two biological replicates. Each data point represents 1-AUC for a PTC organoid line treated by the indicated drug.

**Additional file 3: Figure S3. **Drug response to 13 anticancer agents. PTC organoids were subjected to the targeted drugs vemurafenib, dabrafenib, selumetinib, trametinib, sorafenib, lenvatinib, cabozantinib, vandetanib, and sunitinib, and chemotherapy drugs doxorubicin, vincristine, paclitaxel, and cisplatin. Organoid viability was measured and plotted as percentage of untreated organoids.

**Additional file 4: Figure S4. **Combination drug test of vemurafenib with MEK inhibitors, RTK inhibitors, or chemotherapeutic agents in BRAFV600E-mutant PTC organoid lines. Each data point represents mean ± SEM of 3 independent biological replicates. Organoid viability was measured by CellTiter-Glo assay after 5 days of drug treatment, and results were calculated relative to DMSO-treated control organoids.

**Additional file 5: Figure S5. **The Combination Index (CI) versus Fractional Effect plot for the indicated drug combinations, according to the Chou-Talalay dose-effect method. The CI < 1.0, CI = 1.0, and CI > 1.0 indicate synergism, additivity, and antagonism, respectively.

## Data Availability

All data generated or analyzed during the present study are included in this published article. The raw sequence data of WES reported in this paper have been deposited in the Genome Sequence Archive (Genomics, Proteomics & Bioinformatics 2021) in National Genomics Data Center (Nucleic Acids Res 2022), China National Center for Bioinformation/Beijing Institute of Genomics, Chinese Academy of Sciences (GSA-Human: HRA003468) that are publicly accessible at https://ngdc.cncb.ac.cn/gsa-human.

## Abbreviations

ATC: Anaplastic thyroid cancer
AUC: Area under the curve
BWA: Burrows-Wheeler Aligner
CI: Combination index
CK19: Cytokeratin 19
CNVs: Copy number variations
CTBP: C-terminal binding protein
DAPI: 4’,6-diamidino-2-phenylindole
DMSO: Dimethyl sulfoxide
DTC: Differentiated thyroid cancer
EGF: Epidermal growth factor
EGFR: Epidermal growth factor receptor
ERK: Extracellular signal-regulated kinase
FDA: Food and drug administration
FGF-7: Fibroblast growth factor 7
FGF-10: Fibroblast growth factor 10
FGFR: Fibroblast growth factor receptor
FTC: Follicular thyroid cancer
GATK: Genome analysis toolkit
H&E: Hematoxylin and eosin
HER: Human epidermal growth factor receptor
IC50: Half-maximal inhibitory concentration
MEK: Mitogen-activated protein/extracellular signal-regulated kinase kinase
MTC: Medullary thyroid cancer
NSG: NOD-scid gamma
PDGFRβ: Platelet-derived growth factor receptor-beta
PDOs: Patient-derived organoids
PDX: Patient-derived xenograft
PTC: Papillary thyroid cancer
RAI: Radioactive iodine
RET: Rearranged during transfection
SNVs: Single-nucleotide variants
RTK: Receptor tyrosine kinases
TKI: Tyrosine kinase inhibitor
VEGFR: Vascular endothelial growth factor receptor
WES: Whole-exome sequencing
2D: Two-dimensional
3D: Three-dimensional
