# Supplementary material for: Papillary thyroid cancer organoids harboring BRAFV600E mutation reveal potentially beneficial effects of BRAF inhibitor-based combination therapies
Source: J Transl Med. 2023 Jan 9;21:9. doi: 10.1186/s12967-022-03848-z (PMC9827684; doi:10.1186/s12967-022-03848-z)
Supplement: Supplementary file 1 — Additional file 1: Figure S1. Gene copy number variations of paired PTC organoids and tumor tissues. [file 12967_2022_3848_MOESM1_ESM.docx]

**Additional file 1: Figure S1.** Gene copy number variations of paired PTC organoids and tumor tissues.

**
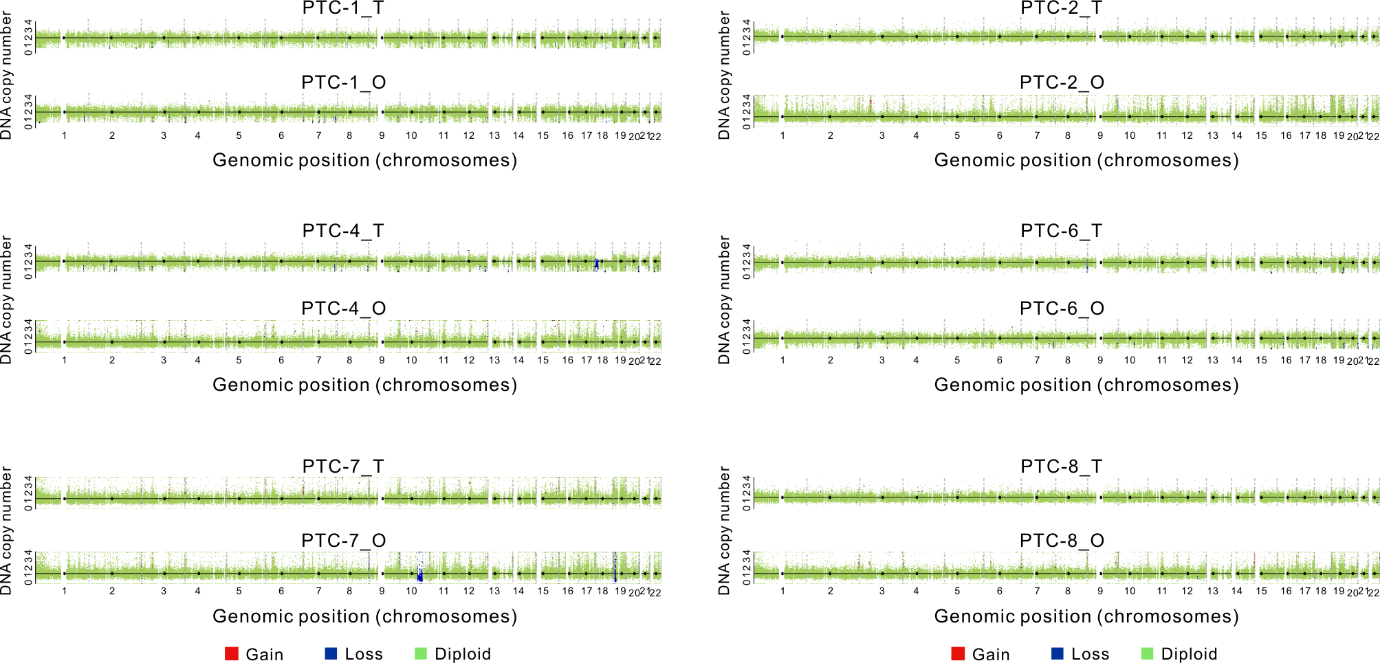
**
