# Supplementary material for: Papillary thyroid cancer organoids harboring BRAFV600E mutation reveal potentially beneficial effects of BRAF inhibitor-based combination therapies
Source: J Transl Med. 2023 Jan 9;21:9. doi: 10.1186/s12967-022-03848-z (PMC9827684; doi:10.1186/s12967-022-03848-z)

**Additional file 2: Figure S2.** Scatterplots of the correlation of 1-AUC values for targeted agents and chemotherapeutic drugs screened by two biological replicates. Each data point represents 1-AUC for a PTC organoid line treated by the indicated drug.


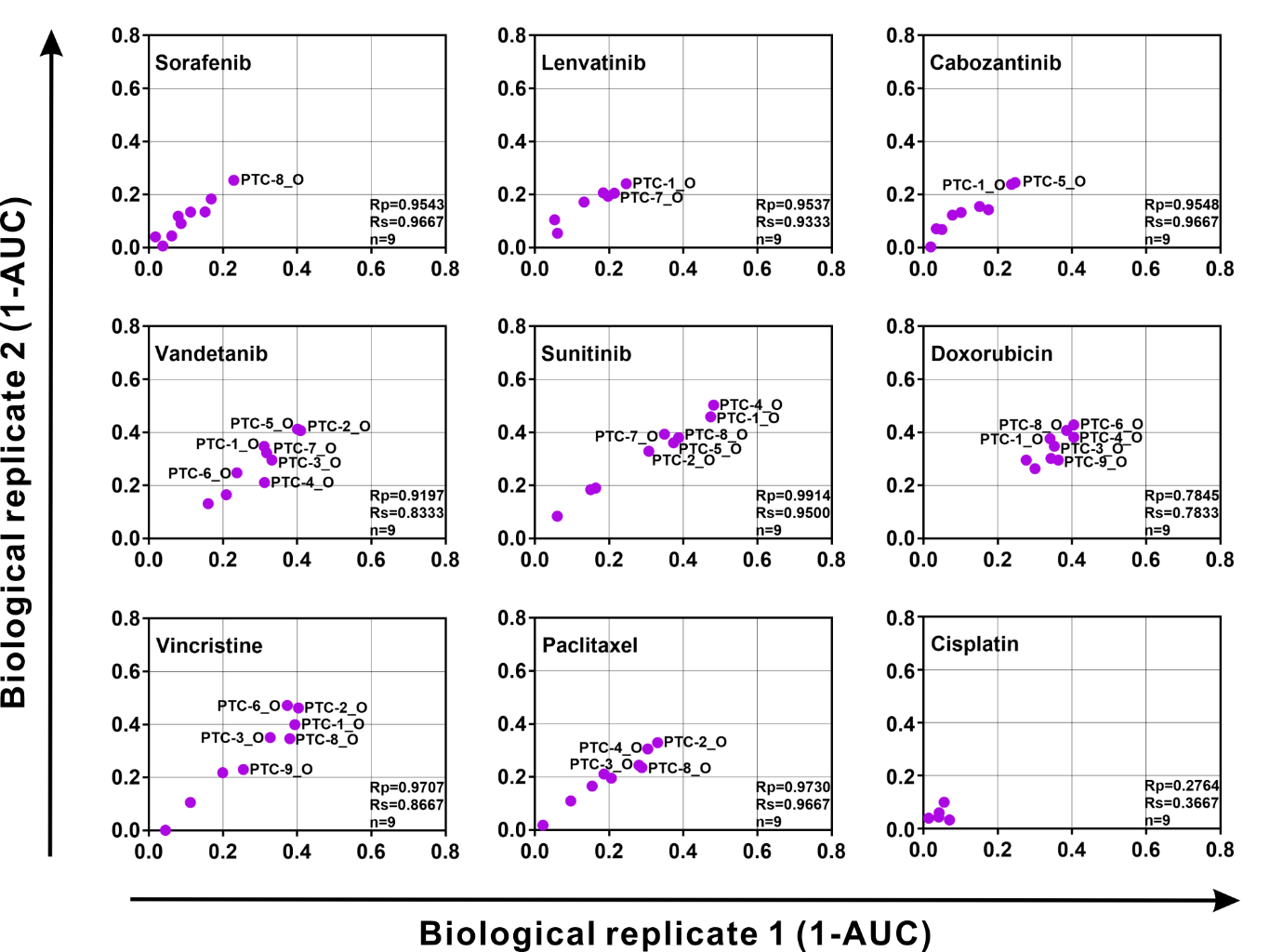

Supplement: Supplementary file 2 — Additional file 2: Figure S2. Scatterplots of the correlation of 1-AUC values for targeted agents and chemotherapeutic drugs screened by two biological replicates. Each data point represents 1-AUC for a PTC organoid line treated by the indicated drug. [file 12967_2022_3848_MOESM2_ESM.docx]
