# Supplementary material for: Papillary thyroid cancer organoids harboring BRAFV600E mutation reveal potentially beneficial effects of BRAF inhibitor-based combination therapies
Source: J Transl Med. 2023 Jan 9;21:9. doi: 10.1186/s12967-022-03848-z (PMC9827684; doi:10.1186/s12967-022-03848-z)

**Additional file 3: Figure S3.** Drug response to 13 anticancer agents. PTC organoids were subjected to the targeted drugs vemurafenib, dabrafenib, selumetinib, trametinib, sorafenib, lenvatinib, cabozantinib, vandetanib, and sunitinib, and chemotherapy drugs doxorubicin, vincristine, paclitaxel, and cisplatin. Organoid viability was measured and plotted as percentage of untreated organoids.


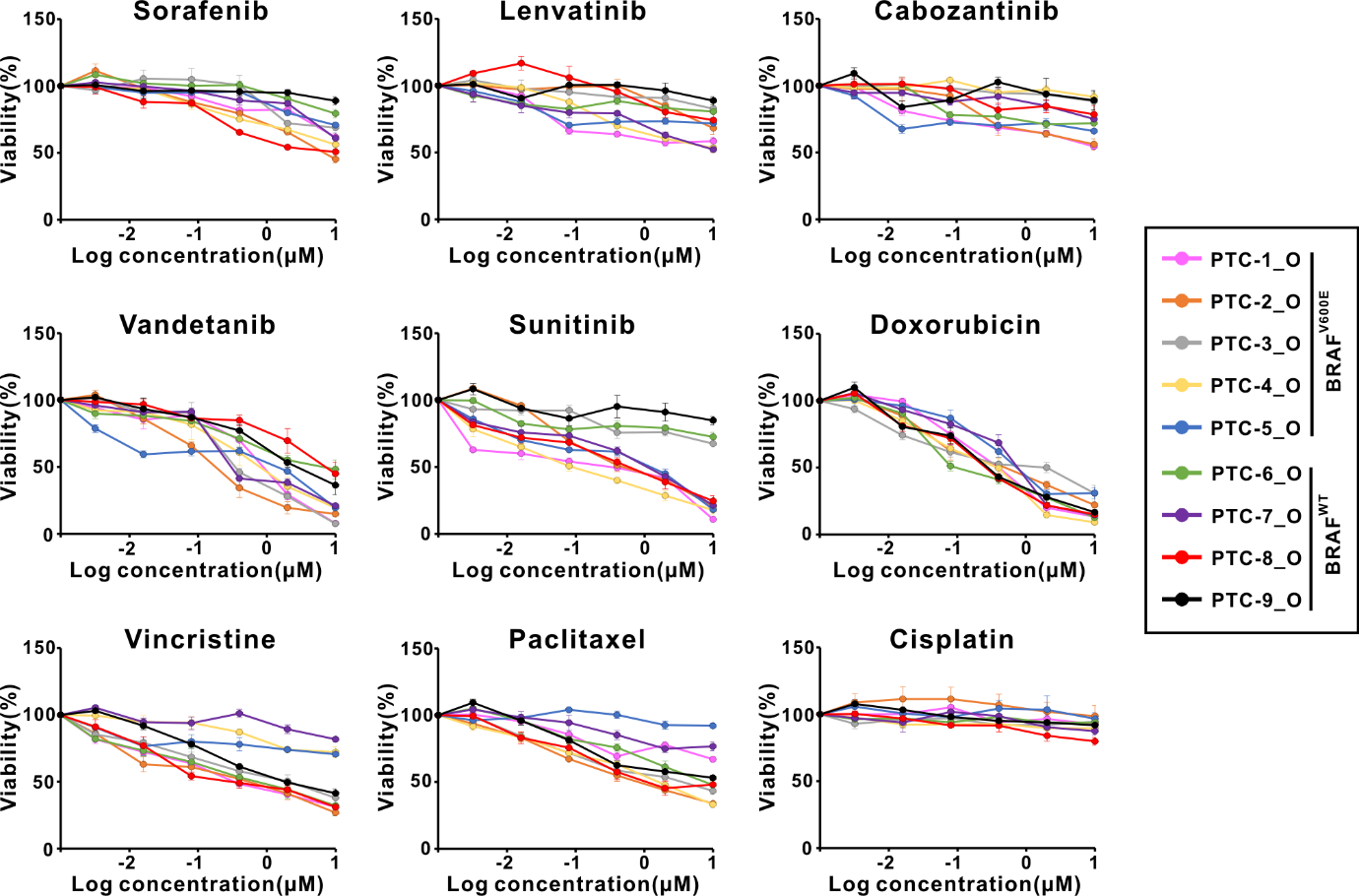

Supplement: Supplementary file 3 — Additional file 3: Figure S3. Drug response to 13 anticancer agents. PTC organoids were subjected to the targeted drugs vemurafenib, dabrafenib, selumetinib, trametinib, sorafenib, lenvatinib, cabozantinib, vandetanib, and sunitinib, and chemotherapy drugs doxorubicin, vincristine, paclitaxel, and cisplatin. Organoid viability was measured and plotted as percentage of untreated organoids. [file 12967_2022_3848_MOESM3_ESM.docx]
