# Supplementary material for: Papillary thyroid cancer organoids harboring BRAFV600E mutation reveal potentially beneficial effects of BRAF inhibitor-based combination therapies
Source: J Transl Med. 2023 Jan 9;21:9. doi: 10.1186/s12967-022-03848-z (PMC9827684; doi:10.1186/s12967-022-03848-z)

**Additional file 4: Figure S4.** Combination drug test of vemurafenib with MEK inhibitors, RTK inhibitors, or chemotherapeutic agents in BRAFV600E-mutant PTC organoid lines. Each data point represents mean ± SEM of 3 independent biological replicates. Organoid viability was measured by CellTiter-Glo assay after 5 days of drug treatment, and results were calculated relative to DMSO-treated control organoids.


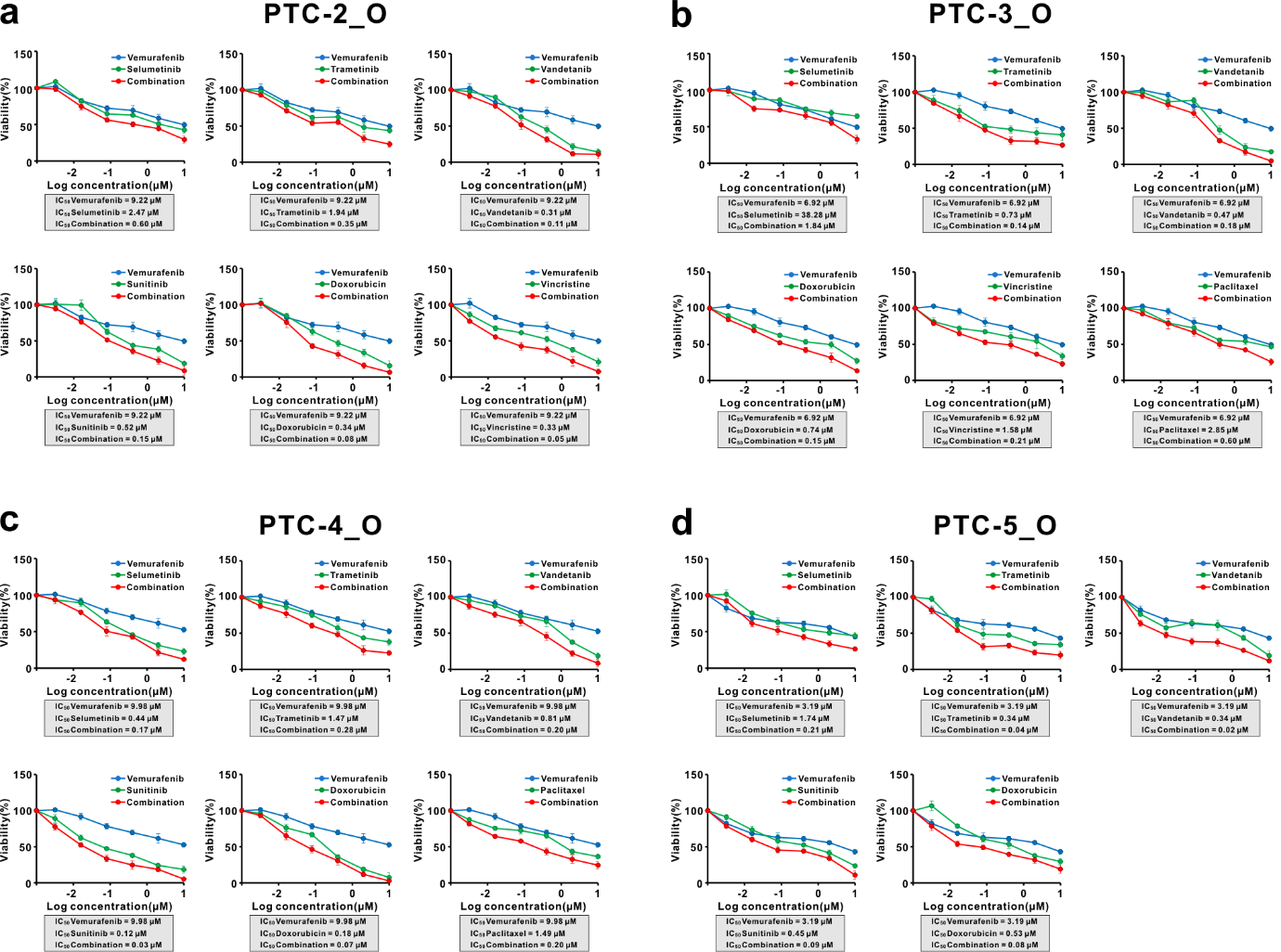

Supplement: Supplementary file 4 — Additional file 4: Figure S4. Combination drug test of vemurafenib with MEK inhibitors, RTK inhibitors, or chemotherapeutic agents in BRAFV600E-mutant PTC organoid lines. Each data point represents mean ± SEM of 3 independent biological replicates. Organoid viability was measured by CellTiter-Glo assay after 5 days of drug treatment, and results were calculated relative to DMSO-treated control organoids. [file 12967_2022_3848_MOESM4_ESM.docx]
