# Supplementary material for: Papillary thyroid cancer organoids harboring BRAFV600E mutation reveal potentially beneficial effects of BRAF inhibitor-based combination therapies
Source: J Transl Med. 2023 Jan 9;21:9. doi: 10.1186/s12967-022-03848-z (PMC9827684; doi:10.1186/s12967-022-03848-z)

**Additional file 5: Figure S5.** The Combination Index (CI) versus Fractional Effect plot for the indicated drug combinations, according to the Chou-Talalay dose-effect method. The CI < 1.0, CI = 1.0, and CI > 1.0 indicate synergism, additivity, and antagonism, respectively.


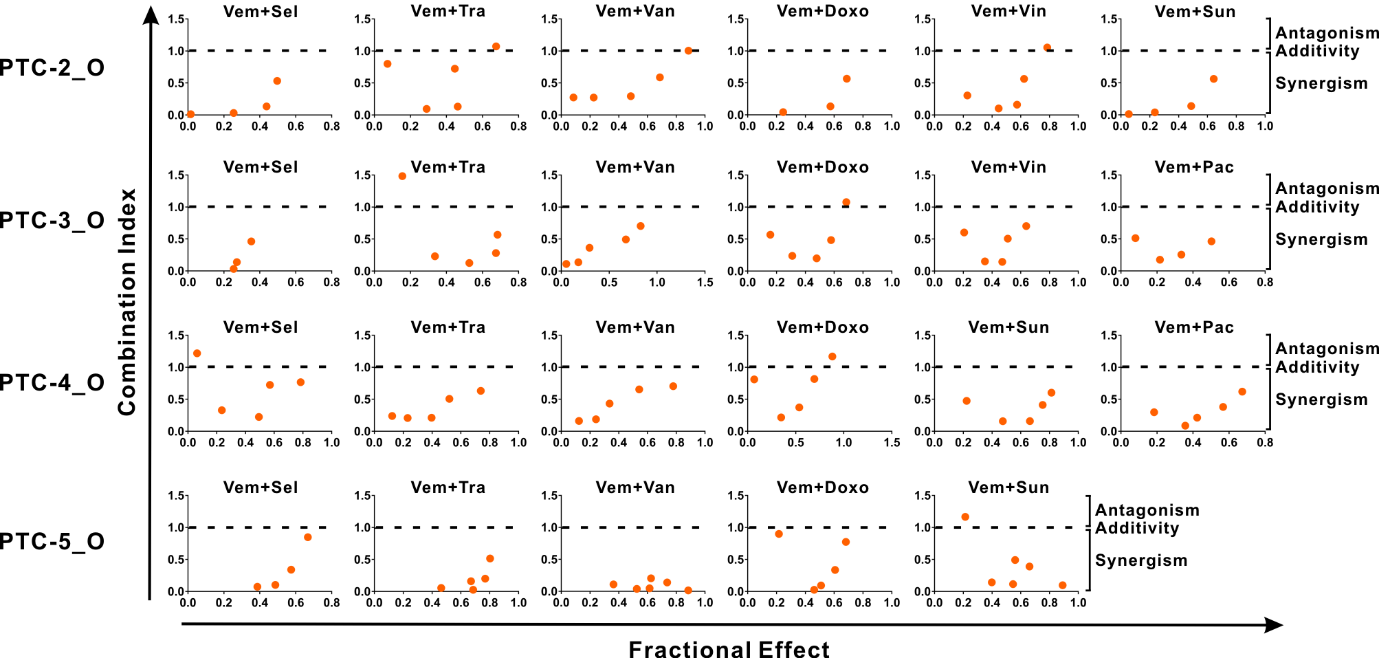

Supplement: Supplementary file 5 — Additional file 5: Figure S5. The Combination Index (CI) versus Fractional Effect plot for the indicated drug combinations, according to the Chou-Talalay dose-effect method. The CI < 1.0, CI = 1.0, and CI > 1.0 indicate synergism, additivity, and antagonism, respectively. [file 12967_2022_3848_MOESM5_ESM.docx]
